# Meiotic behavior and H3K4m distribution in B chromosomes of *Characidium gomesi* (Characiformes, Crenuchidae)

**DOI:** 10.3897/CompCytogen.v10i2.7939

**Published:** 2016-05-18

**Authors:** Érica Alves Serrano, Cristian Araya-Jaime, Elkin Y. Suárez-Villota, Claudio Oliveira, Fausto Foresti

**Affiliations:** 1 Departamento de Morfologia, Instituto de Biociências, Universidade Estadual Paulista, Distrito de Rubião Junior, s/n, 18618-970, Botucatu, São Paulo, Brazil; 2 Instituto de Ciencias Marinas y Limnólogicas, Universidad Austral de Chile, Casilla 567, Valdivia, Chile; 3 Laboratório de Ecologia e Evolução, Instituto Butantan, Avenida Vital Brazil, 1500, CEP 05503-900, São Paulo, São Paulo, Brazil

**Keywords:** Immunodetection, Chromosome painting, SYCP3, synaptonemal complex

## Abstract

*Characidium gomesi* Travasso, 1956 specimens from the Pardo River have up to four heterochromatic supernumerary chromosomes, derived from the sex chromosomes. To access the meiotic behavior and distribution of an active chromatin marker, males and females of *Characidium gomesi* with two or three B chromosomes were analyzed. Mitotic chromosomes were characterized using C-banding and FISH with B chromosome probes. Meiocytes were subjected to immunofluorescence-FISH assay using anti-SYCP3, anti-H3K4m, and B chromosomes probes. Molecular homology of supernumeraries was confirmed by FISH and by its bivalent conformation in individuals with two of these chromosomes. In individuals with three Bs, these elements formed a bivalent and a univalent. Supernumerary and sex chromosomes exhibited H3K4m signals during pachytene contrasting with their heterochromatic and asynaptic nature, which suggest a more structural role than functional of this histone modification. The implications of this result are discussed in light of the homology, meiotic nuclear organization, and meiotic silencing of unsynapsed chomatin.

## Introduction

B chromosomes are genomic structures that are extra to the standard genome set, apparently inert and found throughout animals, plants and fungi (Houben et al. 2014). Although aspects relating to the origin, evolution and function of these chromosomes remain unknown, in some cases they may be derived from autosomes or sex chromosomes (Teruel et al. 2010, Bueno et al. 2013, Pansonato-Alves et al. 2014) or from interspecific crosses (Schartl et al. 1995, McAllister and Werren 1997, Perfectti and Werren 2001). B chromosomes are usually composed of repetitive sequences of DNA, not carrying essential genes (Camacho. 2005, Houben et al. 2014). However, essential gene sequences have been found in some B chromosomes (Banaei-Moghaddam et al. 2013, Ruiz-Estevez et al. 2013, Trifonov et al. 2013), and their presence can generates some phenotypic effects (Houben et al. 2014). During meiosis, B chromosomes show no recombination with A chromosomes, implying a very different evolutionary path (Houben et al. 2014). In addition, B chromosomes are not always found in pairs and do not segregate in a predictable manner during meiosis, which leads to a non-Mendelian segregation in some cases. Even when found in pairs, the transmission rates of B chromosomes are often lower than 0.5 because they are unstable during mitosis and/or meiosis. However, some B chromosomes may show transmission rates higher than 0.5, ensuring their accumulation, an important feature of parasites B chromosomes (Camacho 2000).

The analysis of chromosome pairing during meiosis is a branch of cytogenetic in plants and animals, showing interesting aspects about the chromosome synapsis in the early stages of meiosis (Wallace and Wallace 2003, Basheva et al. 2014, Sanchez-Moran and Armstrong 2014). In particular, the meiotic behavior of B chromosomes has been investigated across different organisms to evaluate aspects of homology, segregation, importance in gamete viability, and transmission to offspring (Fontana and Vickery 1973, Camacho et al. 1980, Jones 1991, Aquino et al. 2013). In *Mazama americana* (Erxleben, 1777), B chromosomes behave as homologous bivalent forms even though they may also occur as univalents, showing erratic behavior responsible for non-Mendelian segregation patterns (Aquino et al. 2013). In fishes bearing four B chromosomes, both the tetravalent conformation and the presence of two bivalents has been witnessed also suggesting homology between these chromosomes. (Pauls and Bertollo 1983, Dias et al. 1998). Meiotic chromosome behavior involves a complex dynamics of chromatin modification playing an essential role in chromosome function and gene regulation (Manterola et al. 2009, Vaskova et al. 2010). In fact, methylation, acetylation, and phosphorylation of the histone nucleosomal core are involved in chromatin assembly, and linked with active and silent transcriptional states during meiosis. For example, the histone H3 methylated at serine 4 (H3K4m) is a epigenetic modification of chomatin that has been linked to gene activation in model organisms, such as mammals (Santos-Rosa et al. 2002, Bernstein et al. 2002, Pokholok et al. 2005, Koina et al. 2009). This modification has been associated to genetic families transcription of barley B chromosomes (Carchilan et al. 2007) and also with X active chromosome in the X-chromosome inactivation process of mammals (Koina et al. 2009). Thus, this histone modification is a good active chromatin marker to analyze B chromosome behavior during meiosis.

The genus *Characidium* Reinhardt, 1867 comprises a group of interesting fish for cytogenetic and molecular studies because of the ZZ/ZW sex determination system (Maistro et al. 1998, Centofante et al. 2001, 2003, Maistro et al. 2004, Noleto et al. 2009), and the occurrence of supernumerary chromosomes (Maistro et al. 1998, Pansonato-Alves et al. 2010, 2011a, 2011b). B chromosomes have been found in Characidium cf. zebra Eigenmann, 1909 (Miyazawa and Galetti Jr 1994, Venere et al. 1999), *Characidium oiticicai* Travassos, 1967, *Characidium pterostictum* Gomes, 1947 (Pansonato-Alves et al. 2010) and *Characidium gomesi* (Pansonato-Alves et al. 2011a), in different levels of heterochromatinization (Pansonato-Alves et al. 2010, 2011b). Individuals of *Characidium gomesi* from the Pardo River posses up to four clearly acrocentric B chromosomes of large size, entirely heterochromatic and originating from the sex chromosomes (Pansonato-Alves et al. 2014). Therefore, supernumerary chromosomes of *Characidium gomesi* represent an interesting model for the study of origin and evolution of these genomic elements in fishes, including chromosome behavior during meiosis, a process less explored in this group of vertebrates.

In this study, we addressed the meiotic behaviour of B chromosomes in specimens of *Characidium gomesi* by means of molecular cytogenetics and immunodetection techniques. B chromosome paint probes and antibodies against synaptonemal complex protein 3 (SYCP3) (Lammers et al. 1994) and against methylated histone H3 at lysine 4 (H3K4) (Pokholok et al. 2005, Godmann et al. 2007, van der Heijden et al. 2007, Kouzarides 2007) were used.

## Materials and methods

### Chromosome preparation

Nine males individuals and seven females of *Characidium gomesi* collected in the Rio Pardo basin of the Rio Paranapanema, Botucatu, São Paulo, Brazil (22°59'25"S and 48°25'40"W) were analyzed (Table 1). The animals were collected in accordance with Brazilian environmental laws of permission to collect issued by MMA / IBAMA / SISBIO, number 3245. The collection procedures, maintenance and analysis of the animals were performed in accordance with international regulations of animal experiments, followed by the Universidade Estadual Paulista (CEEAA / IBB / UNESP protocol number 304). The animals were anesthetized, dissected and mitotic chromosome preparations were obtained following the protocol described by Foresti et al. (1981). The C-banding was performed in mitotic cells according to Sumner (1972). Gonads were removed and processed for SC visualization according to the technique described by Araya-Jaime et al. (2015).

**Table 1.** Individuals of *Characidium gomesi* analysed F: female, M: male.

Ind. nº	Sex	Nº of cells with 0-3 Bs			
		0	1	2	3
75231	M	4	15	3	
75232	M	4	15	5	
75233	F	25			
75234	M	23			
75236	F	7	8	4	
75237	M	8	11	8	
75238	M	3	8	15	
75239	M	8	2	9	
75240	M	1	6	10	
75241	F	2	4	11	12
75242	M	20			
75250	F	6	2	6	17
75251	F	15			
75252	F	5	6	7	
75253	M	17			
75254	F	3	11	17	

### B chromosome painting in mitotic cells

The B chromosome probe was produced as described by Pansonato-Alves et al. (2014) and hybridized in mitotic cells following the procedure described by Pinkel et al. (1986), under high stringency conditions (200 ng probe, 50% formamide, 10% dextran sulfate, 2xSSC at 37 °C overnight).

### Immunodetection and FISH in meiotic cells

Meiotic preparations were immersed for 20 min in 0.01 M citrate buffer preheated to 90 °C. Then, they were incubated in a humidified chamber at 37 °C for 1 h as described by Araya-Jaime et al. (2015). Incubation solutions consisted of rabbit antibodies against SYCP3 of medaka fish (Iwai et al. 2006) diluted 1: 100 in PBS, and mouse antibody against the H3K4m histone of rabbit (Abcam, ab8895) diluted 1: 100 in PBS. After washing in PBS, the slides were subjected to a second incubation in a humid chamber with anti-rabbit donkey IgG conjugated with isothiocyanate fluorescein (FITC; Jackson Immuno Research Laboratories) for detecting SYCP3 and anti-mouse goat IgG conjugated with Texas Red (Invitrogen, Cat No. a-31553) for detecting H3K4m, both diluted 1: 100 in PBS for 1 h at room temperature.

Meiotic preparations were hybridized with the B chromosome probe in accordance with the protocol from Pinkel et al. (1986), adapted by Araya-Jaime et al. (2015) after immunodetection to not affect SYCP3 and H3K4m signals. Because FISH can darken the immunodetection signal, SYCP3 and H3K4m images were captured before the hybridization procedure. Furthermore, the slides were exposed to light before the hybridization to lose the H3K4m signal so that it is not confused with the signals from the B chromosome probe, as this probe was detected with anti-digoxigenin-rhodamine.

Slides were stained with DAPI and mounted with an antifade solution (Vetashield). The images were digitally captured using Image Pro Plus 6.0 software (Media Cybernetics) with appropriate filters of the epifluorescence microscope (Olympus BX61) equipped with an Olympus DP70 camera. Final composition of the images was performed with the application of Adobe Photoshop CS6 image editor software, using image and uniform size scales.

## Results

**Figure 1.**
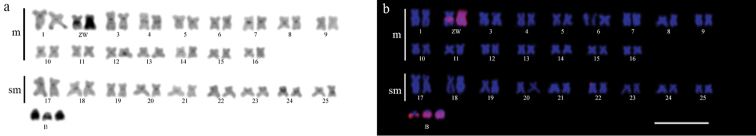
Mitotic karyotype of female *Characidium gomesi* with three B chromosomes. **a** C-banding. Note heterochromatin pattern on B and sex chromosomes **b** Chromosome painting using a B chromosome probe and contrasted with DAPI. Notice probe hybridization on B and sex chromosomes. Bar = 10 μm.

The *Characidium gomesi* specimens showed 2n=50 chromosomes with ZZ/ZW sex chromosomes. In addition, we found individuals with 2–3 acrocentric B chromosomes, with intraindividual number variation (Fig. 1a, Table 1).

All chromosomes of standard complement showed positive C-bands in the pericentromeric region. The W and B chromosomes showed completely dark C-banding, while the Z chromosome showed a large pericentromeric C-band (Figure 1a). Individuals with three supernumerary chromosomes evidenced variation in the amount of heterochromatin between these elements was observed. Chromosome painting with the B chromosome probe demonstrated homology between the B and W chromosomes, as well as with the pericentromeric region of the Z chromosome (Figure 1b).

**Figure 2.**
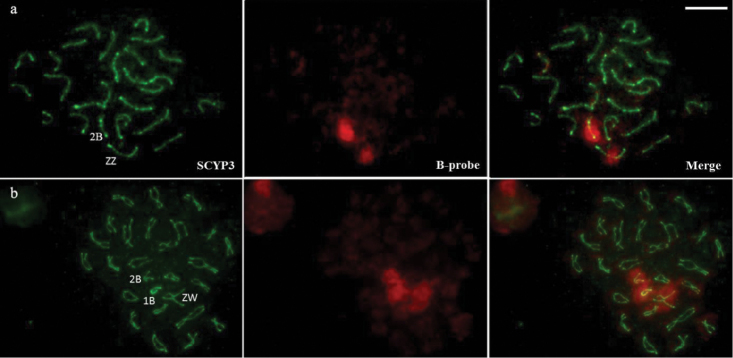
Synaptonemal complex of *Characidium gomesi* after immunodetection with anti-SCYP3 (green) and chromosome painting with B chromosome probe (red). **a** Male pachytene bearing two B chromosomes. Note the positive hybridization of the B paint probe on the bivalents of the B and Z chromosomes **b** Female diplotene bearing three B chromosomes. Note the presence of a B univalent and the ZW bivalent corresponding to the Z and W chromosomes, all positive for the B probe (right). Bar = 10 μm.

**Figure 3.**
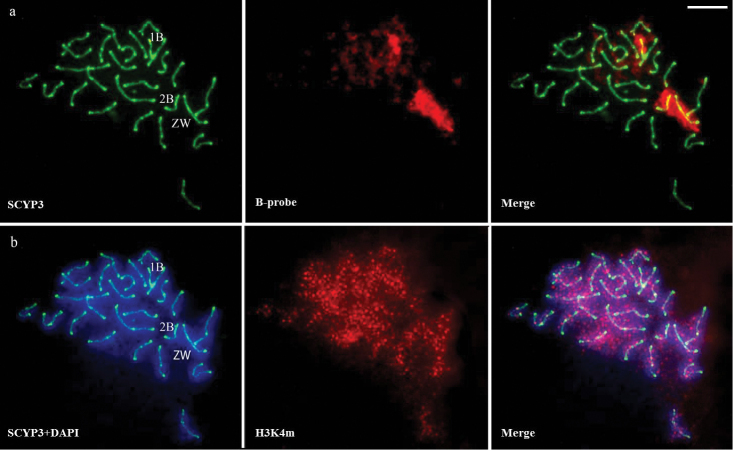
Meiotic cell of *Characidium gomesi* in pachytene stage with three supernumerary chromosomes. **a** Synaptonemal complex protein identified anti-SYCP3 (green) and B chromosome probe (red). Note the positive hybridization of B paint probe on two bivalents and one univalent (ZW, 2B and 1B, respectively) **b** Synaptonemal complex protein identified with anti-SYCP3 counterstained with DAPI (blue), evidencing ZW bivalent and the meiotic formations of B chromosomes; chromatin regions associated with transcriptional activities identified with anti-H3K4m (red) and DAPI consterstaining (blue) evidencing the ZW bivalent and the meiotic formations of B chromosomes. Bar = 10 μm.

Twenty-five bivalents corresponding to the A chromosomes were identified by immunodetection with SYCP3 on cells in the pachytene stage of all individuals. Males and females bearing two B chromosomes exhibited cells with 25 and 26 bivalents (Figure 2a). Additionally, two females with three B chromosomes showed 25 and 26 bivalents, plus an univalent in 50% of the cells (Figure 2b, easily recognized by the bright green signal of SYCP3 and positive hybridization with the B chromosome probe), and in some cells, the subterminal regions of the heteromorphic sex chromosomes showed no synapse during the pachytene. H3K4m immunodetection in pachytene presented a dot-like labeling pattern on autosomes, sex and B chromosomes (Figure 3a). Chromosome painting with the B chromosome probe showed hybridization signals similar to that of mitotic cells (Figure 1b), which corroborates the identification of sex chromosomes (Figures 3b, ZW) and of B chromosomes (Figures 3b, 2B, 1B).

## Discussion

The species of the *Characidium* genus have intriguing cytogenetic features because both sex and supernumerary chromosomes can be found in the same individual (Miyazawa and Galetti Jr. 1994, Vicari et al. 2008, Pansonato-Alves et al. 2010, 2011a). Moreover, in some species, such as *Characidium gomesi*, individuals carry totally heterochromatic B chromosomes (Figure 1), with molecular homology to the sex chromosomes (Pansonato-Alves et al. 2014 and this work). Results of meiotic analysis also demonstrated a perfectly paired bivalent between the two B chromosomes while meiocytes with three supernumerary chromosomes resulted in one bivalent and one univalent of B chromosomes (Figure 2).

Despite sequence homology between B and sex chromosomes in *Characidium gomesi* (Figures 1b, 2, and 3a; see also Pansonato-Alves et al. 2014), the meiotic figures analyzed revealed that the supernumerary chromosomes do not form a multivalent chromosome (Figures 2b and 2a). This recombination restriction with potential donor chromosomes was observed in other organisms and can be considered a starting point in the process of independent evolution of the extra chromosomes (Houben et al. 2014). However, the bivalent conformation of the B chromosomes (Figures 2 and 3) and the results of chromosome painting in mitotic cells (Figure 1b) reinforce the idea of similarity in the supernumerary elements of *Characidium gomesi*. The homology between B chromosomes has been observed in meiotic cells of other fish species, such as *Prochilodus lineatus* (Valenciennes, 1837) (Pauls and Bertollo 1983, Dias et al. 1998, Portela-Castro et al. 2001) and in mammals, such as the American red fox *Vulpes fulvus* (Desmarest, 1820) (Switonski et al. 1987) and the Korean field mouse *Apodemus peninsulae* (Thomas, 1907) (Kolomiets et al. 1988). The formation of a multivalent involving B chromosomes was initially suggested by Pauls and Bertollo (1983) in *Prochilodus lineatus* and later confirmed by Dias et al. (1998). They noted that some meiotic cells of individuals carrying four B chromosomes would either reveal a tetravalent or two bivalents involving the B chromosomes suggesting homology between these chromosomes.

The chromosome pairing seen in meiosis has been interpreted as homology (Ramsey and Schemske 2002) and observed as occurring also between B chromosomes of *Characidium gomesi*. However, such a pairing may occur in a variety of contexts (McKee 2004). In *Drosophila melanogaster* Meigen, 1830 (McKim and Hayashi-Hagihara 1998) and the roundworm *Caenorhabditis elegans* (Maupas, 1900) (Dernburg et al. 1998), these synapses can occur even in the absence of homologous recognition. Conversely, in the F1 of allotetraploid plants, for example, pairing between homologous chromosomes as well as between homeologous chromosomes occur, which are described as partially homologous chromosomes (reviewed in Ramsey and Schemske 2002). As previously described, the chromosome pairing seems to not always indicate complete homology between chromosomes. Nevertheless, the synapse between two B chromosomes of *Characidium gomesi* is considered a crucial condition for chiasmus formation. This formation is mainly composed of physical connections between homologous chromosomes that ensure correct segregation during the first meiotic division (Tsubouchi and Roeder 2003). Furthermore, meiotic recombination caused by the occurrence of crossing-over (Zhang et al. 2014) could be generating a homogenization of the sequences present on both chromosomes; however, a confirmation of late recombination nodules in the B bivalent is required to indicate exchange of genetic material and support this phenomenon.

B chromosomes were detected by hybridization with a probe made from a single supernumerary chromosome (Figures 1b, 2, and 3). However, despite chromosome painting suggested that all supernumerary were composed of similar types of repetitive DNA, one of them did not synapse with the other during pachytene (Figure 2). Even without conclusive evidence about the actual identity of the unsynapsed B chromosomes, the observation of less heterochromatin in one of the supernumerary chromosomes (Figure 1a) could be indicative of the interference of differential heterochromatin composition on the pairing process.

The existence of mechanisms limiting the multivalent pairing between homologous chromosomes was proposed to occur in autopolyploid plants, among which the presence of genetic factors can have a significant effect (see reviewed in Ramsey and Schenske 2002). Therefore, when the B chromosomes carry specific genetic elements, such as the Ph1 gene for example, the pairing of homologous chromosomes may be prevented (Dover and Riley 1972, Kousaka and Endo 2012). When meiosis begins, chromosomes must meet and pair with their counterpart. In this process, the chromosomes are aligned and brought closer, followed by homology recognition and synapsis (McKee 2004, Scherthan et al. 1996, Scherthan 2001). However, this process of meeting between the counterparts during the first meiotic prophase remains largely unknown (Pawlowski and Cande 2005, Da Ines et al. 2014). Thus, the presence of B chromosomes in bivalent and univalent conformation in meiocytes of *Characidium gomesi* appears to depend not only on the similarity of their sequences but also on their meeting and alignment in the meiotic nucleus.

The lack of pairing of one of the B chromosomes in *Characidium gomesi* (Figure 2b and 3) was previously observed in other eukaryotic organisms and also linked to the silencing of genes present in these chromosomal regions. In the fungus *Neurospora crassa* Shear & B.O. Dodge, 1927 (Shiu and Metzenberg 2002) and mice (Turner et al. 2005), chromosomal regions or entire chromosomes not pairing with their counterpart suffer inactivation and induce silencing during meiotic prophase. This process is called meiotic silencing by unpaired DNA (MSUC) and is believed to protect the genome against the invasion of transposable elements (Shiu and Metzenberg 2002). Such silencing was also observed in small unsynapsed regions of trivalents, resulting from multiple Robertsonian translocations (Manterola et al. 2009) and of sex chromosomes, a process known as meiotic sex chromosome inactivation (MSCI). According to these observations, it is expected that the univalent B chromosome of *Characidium gomesi* as well as the unsynapsed region of the sex chromosomes may become inactivated during meiosis since this is a general process observed in other organisms. Studies focusing on transcriptional repression of the B univalent are interesting because they try to confirm this hypothesis.

In mammals, the levels of histone H3 mono-, di- and tri-methylation at lysine 4 is highly dynamic during the development of germ cells. In addition to the possible role of H3K4 methylation in chromatin reorganization, a connection to gene activation has been suggested (Bernstein et al. 2002, Santos-Rosa et al. 2002, Pokholok et al. 2005, Godmann et al. 2007, van der Heijden et al. 2007, Koina et al. 2009). In this study, the presence of histone H3K4m in B chromosomes of *Characidium gomesi* (Figure 3b) disagrees with the heterochromatic nature of these genomic elements (Figure 1b) since this epigenetic marker shows preferential association with euchromatic regions (Godmann et al. 2007, Koina et al. 2009). This incongruence has been noted before in the B chromosomes of barley which simultaneously present subterminal heterochromatic regions enriched with tri-methylated histone H3K4 and repetitive gene families (D1100 and E3900) that are transcriptionally active (Carchilan et al. 2007).

On the other hand, because the lack of synapsis is strongly related to the meiotic silencing by the unpaired DNA process (MSUC) in several organisms (Turner et al. 2005, Burgoyne et al. 2009, Manterola et al. 2009), the presence of H3K4m on the B univalent of *Characidium gomesi* may not necessarily be related to transcription. It is noteworthy that the silencing of telomeres (Nislow et al. 1997, Singer et al. 1998) and of rDNA (Briggs et al. 2001, Bryk et al. 2002) is also related to processes involving histone H3 methylation at lysine 4. Therefore, it can be assumed that the presence of this epigenetic marker in the chromosomes of pachytene stage cells of *Characidium gomesi* may be involved in some of the above processes, in addition to its possible role in the reorganization of chromatin during meiosis.
